# Chemical, Physical, and Biological Corneal Decellularization Methods: A Review of Literature

**DOI:** 10.1155/2024/1191462

**Published:** 2024-03-25

**Authors:** Alexandra Procházková, Martina Poláchová, Jakub Dítě, Magdaléna Netuková, Pavel Studený

**Affiliations:** Department of Ophthalmology, Kralovske Vinohrady University Hospital and 3rd Medical Faculty, Srobarova 1150/50, Prague 10 100 34, Czech Republic

## Abstract

The cornea is one of the most commonly transplanted tissues worldwide. It is used to restore vision when severe visual impairment or blindness occurs in patients with corneal diseases or after trauma. Due to the global shortage of healthy donor corneas, decellularized corneal tissue has significant potential as an alternative to corneal transplantation. It preserves the native and biological ultrastructure of the cornea and, therefore, represents the most promising scaffold. This article discusses different methods of corneal decellularization based on the current literature. We searched PubMed.gov for articles from January 2009 to December 2023 using the following keywords: corneal decellularization, decellularization methods, and corneal transplantation. Although several methods of decellularization of corneal tissue have been reported, a universal standardised protocol of corneal decellularization has not yet been introduced. In general, a combination of decellularization methods has been used for efficient decellularization while preserving the optimal properties of the corneal tissue.

## 1. Introduction

The cornea is a transparent avascular tissue and the main refractive structure of the eye. It enables good visual function with its spherical curvature and transparency, which is secured by highly organised structures and an inner monolayer composed of endothelial cells that control stromal hydration. When corneal transparency is significantly affected (because of a corneal disease or injury), the conservative approach of using topical eye drops is not sufficient; sometimes, the only option to restore visual function is corneal transplantation [1–3].

Corneal transplantation is considered the most frequent transplantation in the world, as corneal blindness is one of the leading causes of blindness worldwide. Unfortunately, there is a global shortage of healthy transplantable human cadaveric donor corneas. A recent global survey quantified that there is only one healthy donor cornea available for the 70 required [4].

In terms of these numbers, it is necessary to develop new alternatives to donor corneas for transplantation. The most promising material appears to be the decellularized corneal tissue itself since it retains the original structure and composition of the native cornea [5, 6]. The decellularization would reduce the immune response and prevent transplant rejection as a cause of graft failure and, therefore, be excellent for corneal transplantation [5–7].

Since the removal of 100% of cell material is difficult to achieve using any widespread decellularization method without compromising the corneal tissue integrity, the minimal decellularization criteria for “sufficient decellularization” that should not lead to an adverse host immune response in vivo has been set as follows: (1) deoxyribonucleic acid (DNA) content: decellularized extracellular matrix should contain less than 50 ng of double-stranded DNA per milligramme of dry weight, (2) the length of the DNA fragment should be less than 200 base pairs, and (3) histological staining with hematoxylin and eosin (H&E) or 4′,6-diamidino-2-phenylindole (DAPI) should show no visible nuclear component within stained tissue [8, 9].

Regarding corneal decellularization, other parameters should also be considered. Every decellularization method can cause an alteration of the extracellular matrix (ECM) and the histoarchitecture of corneas [2, 8]. Transmission electron microscopy (TEM) has been most commonly used to examine the detailed structure of decellularized corneal tissue and its cellular components. It could determine the efficiency of cell removal and preservation of stroma collagen structure. However, fixing protocols required for TEM can alter the collagen fibril structure [2, 9–12]. Scanning electron microscopy (SEM) has been used for the evaluation of the anterior surface of the cornea. Compared to TEM, SEM can be used to visualize larger areas as it is not dependent on transmission, but the extensive dehydratation techniques being used to prepare samples for SEM are associated with a variable amount of shrinkage and can disrupt collagen fibril arrangement. Moreover, harsh fixatives may be required [2, 11, 13, 14]. Concerning evaluation of efficiency of decellularized corneal tissue, a combination of both, TEM and SEM, has been used successfully [9, 11, 13, 15].

Transparency of the cornea is crucial for visual function, and it is another parameter that needs to be examined. Some decellularization methods made the corneas so oedematous that the corneal clarity had to be restored by dehydration using glycerol [16–18]. Transparency can be examined macroscopically or evaluated by light transmittance ranging from 300 to 800 nm wavelengths using a spectrophotometer [13, 19]. Light transmittance can be influenced by corneal thickness that can change after decellularization. Corneal thickness can be measured by optical coherence tomography (OCT) [2, 13]. The biomechanical properties of the decellularized corneal tissue vary depending on a particular decellularization method and its duration of exposure. They can be tested by performing a stretch-stress test using a testing device [8, 19].

Furthermore, it is important to involve in vivo studies (surgical application of corneal tissue) to determine the biocompatibility and efficiency of decellularized corneal tissue and to exclude a negative host immune response leading to functional failure with the need for a new transplant. With no reports of in vivo rejection, xenogeneic scaffolds could be used efficiently for corneal transplantation with the great advantage of their easy accessibility [5, 20–22]. Although sterilisation methods are not within the topic of the current article, it is necessary to mention that they can supplement corneal decellularization methods to ensure maximum effect without rejection after in vivo implantation [23–26].

Concerning new alternatives to donor corneas, several biomaterials have been investigated. There are two main approaches in corneal tissue engineering. The first is the scaffold-based approach that mostly focuses on the production of collagen-based hydrogels. Then, there are other natural polymers and sources that have been investigated (such as silk fibroin, fibrin, chitosan, gelatin, self-assembling peptides, and fish scale). The three-dimensional (3D) bioprinting has been utilized to produce corneal scaffolds using decellularized ECM-based bioinks. Then, there is the second, cell-based approach that focuses on regeneration of the epithelium and endothelium [5, 14, 27–31]. While there were some promising results concerning biomaterials in the studies, there were difficulties in meeting the requirements to be used in clinical practice [9, 28]. Therefore, the use of decellularized corneal tissue as a scaffold should be further investigated as it retains the original structure and composition of the native cornea [5, 6].

In this article, we review different corneal tissue decellularization methods together with their advantages and limitations. The most frequent decellularization methods will be discussed and compared. Finally, we will also discuss possible challenges and future perspectives.

## 2. Methods

When composing this review, we searched PubMed.gov for articles from January 2009 to December 2023 that contained the following keywords: corneal decellularization, decellularization methods, and corneal transplantation. 96 articles were found. These articles were read together with their sources and sorted out. 93 articles were finally used to write this work.

The inclusion criteria to review our topic were a detailed description of different decellularization methods of animal and human corneal tissue followed by appropriate evaluation. In vitro and in vivo studies were included with no minimum number of samples set. Articles without a specific corneal decellularization procedure and its appropriate evaluation were excluded from the work (from results part), as well as articles describing the production of artificial tissues, containing a process of sterilisation, or using cell-based technologies. No article was excluded from work due to a language preference as all articles were available in English.

This article aimed to review different chemical, physical, and biological methods of corneal decellularization and to discuss the best possible method regarding its efficiency and effect on the cornea.

## 3. Results: Corneal Decellularization Methods

### 3.1. Chemical Decellularization Methods

Chemical decellularization methods include surfactants (chemical detergents), acids, bases, and chelating agents. The summary of chemical corneal decellularization methods discussed in this review will be further provided (Table 1).

#### 3.1.1. Surfactants (Chemical Detergents)

Surfactants or chemical detergents lyse cells by disrupting their cellular membranes. They can be ionic with a positive or negative electrical charge, nonionic with no electrical charge, or zwitterionic with zero net charges [51].

*(1) Sodium Dodecyl Sulphate*. Sodium dodecyl sulphate (SDS) is the most widely used ionic detergent because of its effective decellularization properties, with a significant rate of removal of cellular structures and genetic material. Different concentrations of SDS can be used for corneal decellularization, ranging from 0.1% to 2% SDS [18, 35, 51–53].

The use of 0.1% SDS was found to be the most efficient corneal decellularization protocol to decellularize porcine corneas when comparing four different decellularization protocols using benzalkonium chloride (BAK), Igepal, SDS, and Triton X-100. Three different concentrations of SDS, i.e., 0.01%, 0.05%, and 0.1%, were used for 12, 24, and 48 hours [32, 52]. A significant reduction of cells and intact ECM was presented when the protocol of 0.3% SDS and 0.1% ethylene diamine tetraacetic acid (EDTA) was used to decellularize porcine corneas [17, 18]. Successful porcine corneal decellularization was achieved by 0.5% SDS too [54]. A combination of 0.5% SDS, 1% Triton X-100, and nucleases (DNAse and RNAse) was also reported as an efficient method for decellularization of porcine corneas. This protocol was successful in removing DNA and maintaining ECM [2, 55]. Decellularization of bovine corneas using 1% SDS was sufficient and no cellular remains were present in decellularized tissue. However, the DNA concentration in the decellularized tissue was higher compared to samples decellularized by protocols using a solution of trypsin-ethylenediaminetetraacetic acid [12]. To produce an effective hybrid hydrogel for treating corneal wounds, porcine corneal stroma has been decellularized by using a combination of 1% SDS and 2% Triton X-100 with a sufficient decellularization as proven by quantification of DNA (19 ± 3 ng/mg) [14].

0.1% SDS was the most efficient in removing cellular structures and preserving ECM when decellularizing femtosecond-assisted human corneal stromal lenticules compared to other evaluated methods using the hyperosmotic solution, Triton X-100, and nucleases [33].

0.5% SDS kept mechanical strength of decellularized porcine cornea close to the native one [54]. Similarly, 1% SDS preserved mechanical strength of decellularized bovine corneas [12]. However, another study reported that 1% SDS caused changes in corneal mechanical strength, probably due to changes in the structure of the ECM [6].

The optical properties of the decellularized human corneal stromal lenticules with 0.1% SDS were similar to those of the untreated ones [56]. Corneal tissue was highly transparent when a combination of 0.5% SDS, 1% Triton X-100, and nucleases (DNAse and RNAse) was used for porcine corneal decellularization [2, 55]. Transparency of decellularized bovine corneas was lower when 1% SDS was used [12]. The protocol of using 0.3% SDS and 0.1% ethylene diamine tetraacetic acid (EDTA) to decellularize porcine corneas decreased the transparency of the corneas due to swelling. This has been resolved by using 100% glycerol which can almost completely restore corneal transparency [17, 18].

The biocompatibility and safety of decellularized femtosecond-assisted human corneal stromal lenticules using 0.1% SDS were demonstrated by in vivo implanting in rabbit corneal stromal pockets [33]. This procedure could represent the potential for refractive and volume restoration of the cornea [38, 56]. Decellularized human corneal stromal tissue using 1% SDS was successfully implanted in human corneas in vivo to treat advanced keratoconus. The visual acuity of the recipients improved moderately, but with a statistically significant difference. During follow-ups, no clinical inflammation was observed and only a temporary mild lenticule haze was present [34, 57]. However, SDS can induce immunologic reaction, so it must be fully washed after the decellularization process [22].

*(2) Sodium Deoxycholate*. Sodium deoxycholate (SD) is also an ionic detergent used for decellularization. It is not as effective as SDS at the same concentration and incubation time; moreover, it is more disruptive to ECM structure [6].

0.5% and 1% SD could remove all cellular structures of the cornea at an incubation time of 48 hours, but it destroyed the tissue histoarchitecture, specifically collagen fibrils and Bowman's and Descemet's membrane. Decreasing the incubation time to 24 h or 36 h resulted in incomplete removal of the nuclei and structures of corneal cells [6]. The use of 0.5% SD with deoxyribonuclease (DNAse) did not result in a complete removal of cellular material from decellularized human cornea. However, higher concentration of SD resulted in efficient decellularization of human corneas within 24 hours (the use of 1% SD with DNAse). No epithelial and stromal cells were observed using H&E and DAPI staining [7].

*(3) Triton X-100*. Triton X-100 is nonionic chemical detergent used for corneal decellularization. It is less effective in removing cellular structures compared to ionic detergents, but it is also less disruptive. Not all studies documented Triton X-100 to be an efficient method for corneal decellularization [27, 33, 35]. Different concentration of Triton X-100 has been used for corneal decellularization. Whole porcine corneal decellularization protocols using Triton X-100 at three different concentrations (0.01%, 0.05%, and 0.1%) and three different incubation times (12 h, 24 h, and 48 h) were insufficient [32]. An incomplete removal of cells was also reported when using Triton X-100 at a concentration of 0.1-1% to decellularize corneas [6, 35, 39].

Sufficient decellularization of porcine corneas was achieved using 1% Triton X-100 and lyophilization (freeze-drying process). The biocompatibility of the acellular matrix was proved after transplantation to the rabbit corneal stroma without an immune reaction after three months [40]. Efficient decellularization of porcine corneas was achieved by 0.2% Triton X-100 and hypertonic solution (NaCl) [43, 44]. A combination of Triton and NaCl has been even more efficient in decellularizing porcine corneas than the protocol using supercritical carbon dioxide (ScCO2) and NaCl (according to quantification of residual DNA). Moreover, elasticity and strength of Triton-treated corneal scaffold were higher than those treated with ScCO2 [44]. 2% Triton X-100 in combination with ammonium hydroxide or nucleases has also efficiently decellularized corneal tissue (confirmed by H&E and DAPI staining) [41, 42].

*(4) 3-[(3-Chola-Midopropyl) Dimethylammonio]-1-Propanesulfonate (CHAPS)*. 3-[(3-chola-midopropyl) dimethylammonio]-1-propanesulfonate (CHAPS) is a zwitterionic detergent that is not effective enough for corneal decellularization [6, 35]. 0.5% and 1% CHAPS for 24 hours did not present significant removal of cellular structures. Not even an increase in incubation time to 36 hours led to sufficient decellularization but also destroyed the histoarchitecture of corneal tissue [6]. CHAPS had the poorest decellularization effect on corneas compared to SDS and Triton X-100 with a significant number of nuclei remained [35]. These cellular structure remains could lead to an adverse immune response [58].

A combination of CHAPS, SDS, and a nuclease was used to prepare decellularized porcine corneal stroma to produce bioartificial corneas (BACs). A very low DNA content (28.5 ± 5.5 ng/mg) was present after decellularization considering this decellularization to be sufficient. Produced BACs resulted in good biocompatibility and tissue transparency [45]. Therefore, CHAPS can be considered a good complementary decellularization method, but when used alone, it is insufficient.

#### 3.1.2. Acids and Bases

A few protocols for corneal decellularization using acids and bases have been reported [41, 42, 46].

*(1) Acids*. Acids are more commonly used for corneal decellularization than bases [5]. When comparing three different organic acids (formic acid, acetic acid, and citric acid) for porcine corneal decellularization, formic acid was found to be the optimal one, preserving tissue transparency and ECM structure and allowing recellularization potential. Histology analysis also confirmed good decellularization effect on porcine corneas using formic acid. However, acetic acid was unsatisfactory for corneal decellularization [46]. Similarly, the protocol using peracetic acid and ethanol (at different concentrations) to decellularize bovine corneas was unsatisfactory, as cell remains were present in the periphery of decellularized tissue [12].

*(2) Bases*. Only the use of ammonium hydroxide (NH4OH) has been documented in literature for corneal decellularization [5]. 0.1% ammonium hydroxide (NH4OH) together with 2% Triton X-100 was used to decellularize the human corneal stroma. Complete decellularization was confirmed by H&E staining while preserving the structure of the ECM [41, 42].

#### 3.1.3. Chelating Agent

Ethylenediaminetetraacetic acid (EDTA) is a chelating agent usually combined with another decellularization method (such as SDS and trypsin). When used alone, it is ineffective [8, 12, 48–50]. Protocol using a combination of 0.1% EDTA, 0.3% SDS, hypotonic tris buffer, and aprotinin was reported to decellularize porcine corneas. After decellularization, H&E and DAPI staining confirmed significant but incomplete cell removal from the stroma maintaining the ECM structure [17]. A very similar result was obtained after decellularization of bovine corneas using 0.1% EDTA, aprotinin, and 0.3% SDS [18].

A good decellularization effect to decellularize bovine corneas was achieved by a solution of trypsin-ethylenediaminetetraacetic acid (TE). There were no cell remains and the overall structure was maintained. Moreover, the transparency of the decellularized tissue was not affected [12]. The hypotonic solution of 0.5% TE was used to completely decellularize human corneal lenticules. DNA content met the requirements for sufficient corneal decellularization while maintaining ECM structure. This solution was more efficient than decellularization protocols using 0.5% SDS and 0.5% Triton X-100. Moreover, hypotonic TE-treated decellularized human corneal lenticules were sufficiently transparent and biocompatible [48].

### 3.2. Physical Decellularization Methods

Physical methods used for corneal decellularization are freeze-thaw cycles, hypotonic and hypertonic solutions, high hydrostatic pressure (HHP), ultra-high hydrostatic pressure (UHHP), and supercritical carbon dioxide (scCO2). The summary of physical corneal decellularization methods discussed in this review will be further provided (Table 2).

#### 3.2.1. Freeze-Thaw Cycles

Repeated freeze-thaw cycles (−80°C for freeze and 37°C for thaw) can be used for the decellularization process by lysing cells while keeping the structure of the extracellular matrix with only minimal disruptions and good optical properties. Usually, five to six freeze-thaw cycles have been used for corneal decellularization [8, 27]. Porcine corneas were decellularized by SDS, Triton X-100, and freeze-thaw cycles. All hydrogels produced from ECM of decellularized corneas were highly transparent, but those decellularized by freeze-thaw cycles were with the best transparency. Staining with H&E confirmed no cell nuclei present after decellularization [27, 51]. Porcine corneas were decellularized in combination with freeze-thaw cycles lysing cells and the nuclease destroying residual DNA. No nuclei were present after decellularization [59]. However, this decellularization method is ineffective in removing cells and genetic content on its own, so it is efficiently used in combination with other techniques (nucleases or chemical detergents) [8, 27, 59, 67].

#### 3.2.2. Hypotonic and Hypertonic Solutions

Hypotonic and hypertonic solutions effectively lyse cells and help rinse cellular remains but not sufficiently enough. To achieve better decellularization outcomes, they can be used in combination with nucleases (such as DNAse and RNAse) which effectively remove nucleotides after cell lysis [8, 39].

Hypertonic solutions (mostly sodium chloride (NaCl) at a concentration of 1.5 M) have been successfully reported to remove cell material while maintaining ECM components [33, 60, 68]. A very good decellularization result was achieved when NaCl-prepared corneas were washed with 0.2% Triton X-100 [43, 44]. While using five different decellularization methods to decellularize human corneas, Triton X-100, poly (ethylene glycol) (PEG), and liquid nitrogen were not sufficient to remove all the cellular material. On the contrary, corneas treated with SDS or NaCl plus nucleases were efficiently decellularized and resulted in complete removal of the cellular material. However, only the 1.5 M NaCl plus nuclease protocol (DNAse and RNAse) left the epithelial basement membrane of the corneas completely intact and close to that of the untreated ones [39]. Due to the great result, the previously mentioned successful protocol was adopted for decellularization of human corneas. Complete corneal decellularization was achieved using NaCl plus nucleases. H&E and DAPI staining confirmed sufficient removal of cellular structures, while fibrillar structures remained unchanged [42]. NaCl with nucleases were also efficiently used to decellularize whole human corneas while preserving the structure of the ECM [36]. 1.5 M NaCl and DNase were efficiently used to decellularize small-incision lenticule extraction (SMILE) lenticules as proven by histological staining and DNA quantification. However, some disruption in ECM structure could be noted [61]. Similarly, 1.5 M NaCl was successfully used to decellularize porcine corneas while corneal decellularization with 0.1% SDS was insufficient [60].

However, there were also studies reporting inefficient corneal decellularization by NaCl. According to these studies, NaCl was inefficient in removing cellular material and left the highest amount of residual DNA compared to other decellularization methods (SDS and Triton X-100) [37, 47].

#### 3.2.3. High and Ultra-High Hydrostatic Pressure

High hydrostatic pressure (HHP) used for decellularization has become increasingly widespread. This method uses pressure greater than 600 MPa to destroy cell membranes [20, 51]. HHP and ultra-high hydrostatic pressure (UHHP) were successfully used to decellularize porcine corneas with complete removal of cells while maintaining the ECM structure. In addition, produced scaffolds were proved to be noncytotoxic after transplantation into rabbit corneas [62, 63]. The great advantage of this method is the possibility of solitary application [51]. However, it has not been widely used due to the expensive equipment required [53, 58].

#### 3.2.4. Supercritical Carbon Dioxide

Supercritical carbon dioxide (scCO2) is being used as an alternative technique for decellularization. It bursts cells with high fluid pressure and effectively removes them from the tissue by rapid depressurisation. ScCO2 significantly reduces the decellularization time while maintaining the extracellular structure of corneas comparable to natives. It can be used simultaneously to sterilize tissue by obtaining immunocompatible acellular scaffolds. The limitation of this method is the complex scCO2 reactor system needed [44, 64, 65].

ScCO2 has been used to decellularize bovine corneas with an effective cell removal confirmed by H&E staining. The control group decellularized with 1% SDS was inferior to the scCO2 decellularization method in terms of ultrastructure maintenance [64]. Complete corneal decellularization was also reported after combining a 1-hour scCO2 decellularization method followed by a washing process. However, this combination resulted in an increased gap between collagen fibrils that changed a little of the stromal layer [65].

ScCO2 was also successfully used to produce acellular porcine corneas. A complete decellularization has been confirmed by histological staining and DNA quantification. Treated tissue was transplanted into rabbits with good biocompatibility [44, 66].

### 3.3. Biological Decellularization Methods

Biological methods of corneal decellularization include enzymes or serum. The summary of biological corneal decellularization methods discussed in this review will be further provided (Table 3).

#### 3.3.1. Enzymes

Enzymes have been used efficiently for corneal decellularization. They help remove nucleotides after cell lysis, but they are unable to remove cellular remains on their own. Therefore, they must be used with another decellularization method [8, 13, 34, 42, 50].

Nucleases (DNAse and RNAse) are enzymes that have been efficiently used for corneal decellularization. They significantly reduced DNA material and also caused the treated tissue to be less transparent. A better decellularization result was achieved after pretreatment with a chemical agent or another enzyme [7, 37]. The human corneas unsuitable for keratoplasty were decellularized with a combination of 1% SDS and DNAse. This combination was effective in removing all cellular structures while preserving the alignment of collagen fibrils [47]. 1% SDS with DNAse was also efficient in decellularizing human corneas [7]. A combination of nucleases and chemical detergents (0.5% SDS and 1% Triton X-100) has successfully decellularized porcine corneas [2, 55]. Human corneas were efficiently decellularized using NaCl plus nucleases. All cellular structures were removed. Corneal decellularization using only NaCl was insufficient with cellular remains present in the stroma [36]. A combination of NaCl and DNAse was efficiently used to decellularize SMILE lenticules, as proven by histological staining and DNA quantification. However, some disruption in ECM structure could be noted [61]. Freeze-thaw cycles were used to decellularize porcine corneas followed by nuclease treatment to remove residual nuclei from corneal tissue. Nucleases are often used in decellularization protocol to remove residual genetic material [69, 72].

Phospholipase A2 (PLA2) is also an enzymatic agent used for corneal decellularization. PLA2 in combination with 0.5% SD was efficiently used for porcine corneal decellularization with sufficient removal of genetic material while preserving 80% glycosaminoglycan (GAG) compared to native porcine corneas. The scaffolds produced by this method remained with optical and mechanical properties and without significant change. Biocompatibility with no rejection was evaluated 12 months after animal transplantation (rabbit lamellar keratoplasty) [73].

Trypsin is an enzyme efficiently used for corneal decellularization in combination with EDTA. This solution resulted in sufficient decellularization of corneal tissue with the overall structure maintained, also with the good transparency and biocompatibility. The solution of 0.5%  TE was even more efficient in corneal decellularization than protocol using 0.5% Triton X-100 [12, 48].

Supernuclease and mild detergent sodium N-lauroyl glutamate (SLG) has been efficiently used for rapid decellularization of porcine corneal stroma. Supernuclease is a homologous nuclease of benzonase not often used for corneal decellularization. However, decellularization by supernuclease and SLG has resulted in complete removal of nuclei as proven by DAPI staining. Efficiency of this decellularization method was also confirmed by DNA quantification. Moreover, decellularized corneas were almost transparent and the alignment of collagen fibrils was regular [13].

#### 3.3.2. Serum

The use of serum can be another efficient corneal decellularization method. Bovine serum for 72 hours has been efficiently used to decellularize SMILE lenticules as proven by histological staining and DNA quantification. No alteration of the arrangement of the collagen fibrils or transparency has been present. Bovine serum for 72 hours was more effective in decellularization of SMILE lenticules than combination of NaCl and DNAse [61].

Human serum has been efficiently used with electrophoresis for corneal stromal tissue decellularization to obtain a good biocompatible scaffold. The decellularized corneal tissue obtained by this method showed optical, biomechanical, and ultrastructural similarities to those of native corneas. In vivo studies did not show rejection after lamellar keratoplasty (LKP). However, incubation with human serum itself would not have sufficiently removed the cellular structures, so electrophoresis had to be used [53, 71]. The advantage of this method is that no additional enzymes or harmful detergents are needed. Moreover, the good biocompatibility, no immunogenicity, and good transparency of transplanted corneas have been proved [70].

### 3.4. Efficiency and Overview of Corneal Decellularization Methods

**Figure 1:**
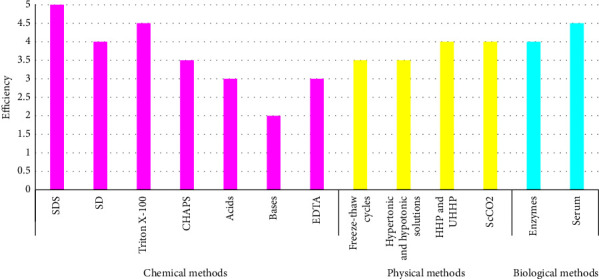
The summary of efficiency of corneal decellularization methods (the most efficient decellularization method represents number “5” and the least efficient decellularization method goes to number “0”).

**Figure 2:**
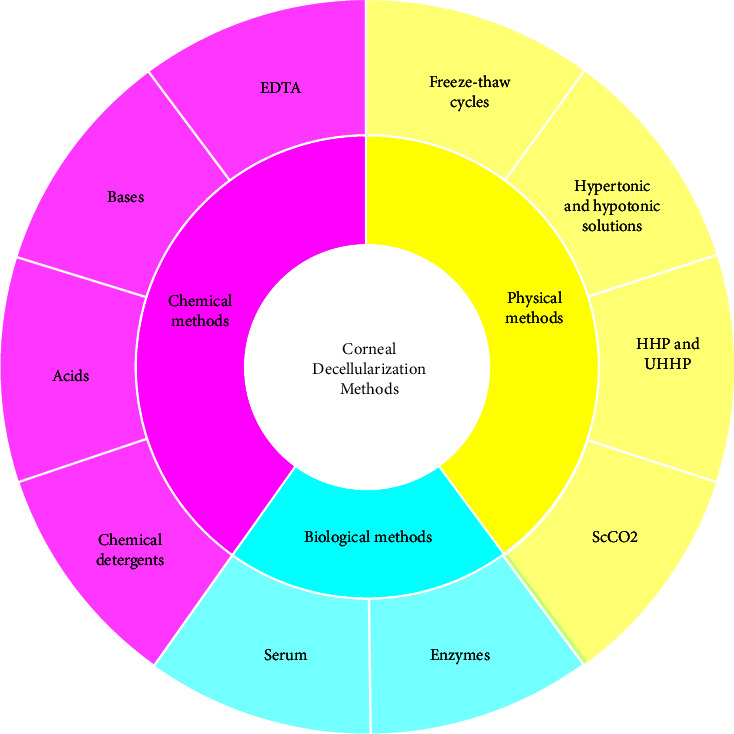
The overview of corneal decellularization methods.

Different chemical, physical, and biological decellularization methods have been reviewed. Evaluation of efficiency of each decellularization method (Tables 4–6) together with the summary (Figure 1) has been provided. Efficiency of corneal decellularization methods was evaluated according to criteria set for “sufficient decellularization” (mostly DNA quantification and histological analysis with H&E and DAPI staining) documented in available articles [8]. These articles were listed in the column named “References” (Tables 4–6). However, it was quite challenging as there are many decellularization methods and often their combination has been used with different concentrations and incubation times. For better visualization, an overview of corneal decellularization methods has been drawn (Figure 2).

## 4. Discussion

Decellularized corneal tissue appears to be a promising scaffold for corneal transplantation, as sources of human donor corneas are limited. This is supported by the fact that the requirement for corneal transplantation is increasing.

Several successful techniques of xenogeneic corneal decellularization have already been described. Corneas of different species of animals (pig, cow, ostrich, rabbit, squid, dog, and cat) have been successfully used for corneal decellularization to construct the tissue-engineered corneal scaffolds [5, 12, 23, 24, 35, 54, 72, 74]. Porcine corneas have been the most commonly used for corneal decellularization mainly due to their ease of availability and multiple anatomical and physiological similarities with human corneas. All five layers of the cornea (epithelium, Bowman's layer, stroma, Descemet's membrane, and endothelium) are histologically visible in pigs. Porcine corneal endothelium is composed of a monolayer of endothelial hexagonal cells (just like human endothelium), ensuring good corneal transparency and vision [5, 31, 75, 76]. The histology of canine corneas has shown some similarities with humans; however, there are some key differences (for example, a layer comparable to Bowman's layer in humans does not exist in dogs) [77]. Anatomical structure of the ostrich cornea (thickness of the central cornea, refractive power, and number of cell layers of central corneal epithelium) is closer to human cornea compared to porcine cornea [24]. Porcine corneas were found structurally closer to human corneas compared to rabbit corneas considering corneal interlamellar distance. Different studies have also had different sights regarding the presence and the absence of the Bowman's membrane in rabbits [35, 76]. Furthermore, the immunogenicity of porcine corneal tissue is much lower than that of other species [6, 78]. The use of porcine corneas does not represent ethical issues as these are usually considered as waste in food industry. Therefore, they are also much more cost effective [21, 75, 78]. Considering all aspects and numerous studies, porcine corneas seem to be the most suitable substitute animal material for human corneas [14, 45]. This is supported by the fact that acellular porcine corneas (APCs) have been already successfully used in clinical research to treat patients with corneal diseases. Acellular porcine corneal stroma applied to patients was confirmed to be effective and safe [15, 79, 80]. However, decellularized human cadaveric corneas are an even better option compared to animal corneas as there is no risk of zoonotic disease [81]. A potential source for a partial corneal transplantation could be corneas with nonviable endothelium and the corneal remains of posterior LKP or femtosecond laser-assisted refractive lenticule extraction [33, 56, 57, 82]. Another potential source could be human lens capsules that were efficiently decellularized and used for endothelial tissue engineering [49].

The creation of an optimal corneal decellularization protocol could help solve the problem of human donor corneal shortage. It could also simplify the storage and extend the availability of corneal tissue in developing countries with limited access to eye banking. Efficiently decellularized corneal tissue could help treat corneal diseases (keratitis, ectasia, and perforations) or correct hyperopia and presbyopia [26, 34, 82]. The main challenge to corneal decellularization is to “sufficiently decellularize corneas” while maintaining their transparency, ECM structure, and biocompatibility [2, 8, 39, 58].

Today, corneas are mainly decellularized by chemical methods, with detergents being the most common one. They are very effective in removing cellular structures and genetic material [39, 53, 72]. Therefore, they are also often used in decellularization of other tissues. Rat aorta was efficiently decellularized by 0.5% SDS. Even more efficient was a combination of 0.25% SDS and 0.5% Triton X-100. Bovine lungs were efficiently decellularized by 1% SDS and DNAse. Combination of 1% Triton X-100 for 72 hours and DNAse for 1 hour was also effective in decellularization of bovine lungs, but protocol with SDS was more efficient [83, 84]. Hypotonic and hypertonic solutions have been effectively used for corneal decellularization in combination with other decellularization methods [39, 43, 44]. The same applies to freeze-thaw cycles and enzymes that have often been used for efficient corneal decellularization but in combination with other methods [2, 36, 37, 53]. The advantage of the aforementioned methods is low cost and easy accessibility. The disadvantages are possible changes in ECM structure and decreased transparency of corneal tissue due to swelling. Therefore, a combination of decellularization methods has been used mainly to achieve “sufficient decellularization” with less damage to the cornea. Strong and weak decellularization methods could complement each other [20, 51, 53, 55]. Human serum in conjunction with electrophoresis has been also reported to be an efficient decellularization method with no need for additional enzymes or chemical detergents to ensure biocompatibility and maintain transparency [70, 71]. High or ultra-high hydrostatic pressure can decellularize the corneas on their own. Their other advantage, together with supercritical carbon dioxide, is a short treatment time. The negative is the expensive equipment needed [20, 46, 51, 53, 85].

The progress in the field represents production of hydrogels and biomaterials produced by cell-based technologies and 3D bioprinting. However, their limitation represents clinical application [14, 19, 27, 29, 51]. Some acellular corneas have entered clinical research as a potential alternative for human donor corneas for treatment of corneal diseases. Acellular porcine corneal stroma (APCS) has been used in clinical studies to treat patients with various types of corneal infections. A combination of physical (NaCl) and chemical (0.2% Triton X-100) decellularization methods together with sterilization (Co^60^ radiation) has been used to produce safe and effective APCSs for human keratoplasty [79, 86]. Acellular porcine corneal lenticules (Xenia implants) have been efficiently used in patients for treatment of advanced keratoconus and ectasia. A retrospective clinical study documented their safety and effectiveness. After extraction from porcine tissues, lenticules were decellularized, cross-linked, and sterilized before further use [80, 87]. Gamma-irradiated sterile corneal tissues (VisionGraft™) have been efficiently used for corneal and glaucoma patch surgeries when viable endothelium was not needed. Corneal tissue underwent irradiation dose from 17 to 23 kGy according to a patented technology to produce a sterile acellular corneal tissue [88, 89]. The other clinical studies documented safety and efficacy of corneal stromal lenticules (CSLs) obtained by SMILE refractive surgery for the treatment of corneal ulcers and perforations. These acellular corneal tissues were cryopreserved at −80°C from 2 to 6 months before transplantation [90–92]. Decellularized corneal stromal laminas obtained from human donor corneas with nonviable endothelium were used in a clinical trial to evaluate safety and efficiency in patients with advanced keratoconus. Corneal stromal laminas were decellularized by a combination of decellularization methods (1% SDS and DNAse). Their implantation in vivo was documented to be safe and moderately effective for the treatment of advanced keratoconus compared to classical corneal transplantation techniques [34, 93]. At the moment, there are probably other ongoing clinical trials concerning acellular corneas with no data published yet. With further research, there is a great probability of creating the best possible alternative to human donor corneas to help resolve their shortage and treat corneal diseases.

## 5. Conclusion

Chemical, physical, and biological corneal decellularization methods were compared according to the recent available literature. In last few years, there has been a great progress in developing decellularized biomaterials for the treatment of corneal diseases. Most promising are xenotransplants and hydrogels, where a combination of decellularization methods has been used. Among decellularization methods, mostly chemical detergents have been used for their high efficiency and easy availability. The biomaterials produced by cell-based technologies and 3D bioprinting also attract great attention. These methods represent new promising approaches in production of corneal tissue replacements. Despite the great progress in producing corneal tissue transplant, there are still challenges that need to be resolved including clinical application and potential development of full thickness donor cornea for the treatment of corneal diseases.

## Figures and Tables

**Table 1:** The summary of chemical corneal decellularization methods discussed in this review.

Chemical decellularization methods		Advantages/disadvantages	Significance for future research	References
Surfactants	SDS	(i) Very efficient in removing cellular structures and genetic material (ii) Can induce immunologic reaction; must be fully washed after decellularization process (iii) Can cause changes in the ECM structure (iv) Transparency can be lower at higher concentration	(i) High	[6, 14, 17, 22, 32–38]
	SD	(i) Effective decellularization method (ii) Less effective than SDS (often used with other methods, mostly nucleases) (iii) Disruptive for collagen fibrils (less than SDS)	(i) Medium	[6, 7]
	Triton X-100	(i) Not all studies documented sufficient decellularization of corneal tissue (ii) For an efficient corneal decellularization, must be used in combination with other decellularization methods (iii) Less effective than ionic detergents (SDS and SD) but also less disruptive (iv) Can cause minimal changes in ECM structure	(i) Medium/high	[2, 27, 33, 35, 37, 39–44]
	CHAPS	(i) Ineffective for corneal decellularization when used alone (ii) Effective when used with another decellularization method (such as SDS) (iii) Destruction of histoarchitecture of corneal tissue at longer incubation time	(i) Low/medium	[6, 35, 45]

Acids		(i) Not all studies document sufficient corneal decellularization (ii) Slight changes in ECM structure	(i) Low/medium	[12, 46]

Bases		(i) Only the use of ammonium hydroxide (NH4OH) has been documented for corneal decellularization in the literature (ii) Need to be used with another effective decellularization method	(i) Low	[5, 41, 42]

EDTA		(i) Incomplete cell removal when used alone (ii) Efficient decellularization when combined with another decellularization method (for example, SDS and trypsin)	(i) Medium	[8, 12, 17, 18, 47–50]

**Table 2:** The summary of physical corneal decellularization methods discussed in this review.

Physical decellularization methods	Advantages/disadvantages	Significance for future research	References
Freeze-thaw cycles	(i) Good corneal decellularization effect by lysing cells (ii) Need to be used in combination with other decellularization techniques to remove cells (iii) Disruption to ECM structure (iv) Better optical properties of decellularized corneal tissue compared to the use of chemical detergents	(i) Medium	[8, 27, 59]

Hypertonic and hypotonic solutions (mostly NaCl)	(i) Efficient decellularization achieved when in combination with other decellularization method (for example, with nuclease or Triton X-100) (ii) Minimal disruption in the ECM structure (iii) Good optical properties (iv) Less sufficient compared to chemical detergents	(i) Medium	[33, 36, 37, 39, 42–44, 60, 61]

High or ultra-high hydrostatic pressure (HHP, UHHP)	(i) Efficient decellularization (ii) Minimal changes in the ECM structure (iii) Solitary application (iv) Expensive equipment required	(i) Medium	[51, 62, 63]

ScCO2	(i) Effective corneal decellularization (ii) Reduces decellularization time (iii) Can cause changes in ECM structure (increase gap between collagen fibrils) (iv) Can be simultaneously used to sterilize corneal tissue (v) Less expensive compared to HHP (vi) Limitation is the need of the complex scCO2 reactor system	(i) Medium/high	[44, 64–66]

**Table 3:** The summary of biological corneal decellularization methods discussed in this review.

Biological decellularization methods	Advantages/disadvantages	Significance for future research	References
Enzymes	(i) Often used in decellularization protocols to help remove residual genetic material after cell lysis (ii) Must be used with another decellularization method (iii) Can disrupt ECM structure (iv) Less transparent decellularized corneal tissue	(i) High	[7, 8, 34, 36, 37, 42, 47, 50, 55, 61, 69]

Serum	(i) Efficient decellularization method (ii) Maintained ultrastructure of decellularized corneal tissue (iii) Good optical properties after decellularization (iv) No need for other decellularization methods	(i) High	[61, 70, 71]

**Table 4:** The evaluation of efficiency of chemical decellularization methods on corneal tissue and assays used for the evaluation.

Chemical decellularization methods		Decellularization efficiency	Assays used for determination of efficiency of corneal decellularization	References
Surfactants	SDS	+++++	Histological analysis with H&E and DAPI staining	[6, 7, 12, 18, 22, 32, 33, 35, 37, 38, 52]
	SD	++++	Histological analysis with H&E staining	[6, 7],
	Triton X-100	++++/+++++	Histological analysis with H&E and DAPI staining	[2, 35, 37, 39, 41–44]
	CHAPS	+++/++++	Histological analysis with H&E staining, DNA quantification	[6, 35, 35, 45]

Acids		+++	Histological analysis with H&E staining, DNA qualification	[12, 41, 42, 46]

Bases		++	Histological analysis with H&E staining	[41, 42]

EDTA		+++	Histological analysis with H&E and DAPI staining, DNA quantification	[12, 17, 18, 47, 48]

*Note*. For evaluating efficiency, we used the “+” symbol, where + is the least efficient decellularization method and +++++ is the most efficient decellularization method.

**Table 5:** The evaluation of the efficiency of physical decellularization methods on corneal tissue and assays used for the evaluation.

Physical decellularization methods	Efficiency	Assays used for determination of efficiency of corneal decellularization	Sources
Freeze-thaw cycles	+++/++++	Histological analysis with H&E and DAPI staining	[27, 59]
Hypertonic and hypotonic solutions (mostly NaCl)	+++/++++	Histological analysis with H&E and DAPI staining, gel electrophoresis, quantification of DNA	[33, 36, 37, 39, 42–44, 47, 60, 61]
High or ultra-high hydrostatic pressure	++++	Histological analysis with H&E staining, quantification of DNA	[62, 63]
ScCO2	++++	Histological analysis with H & E and DAPI staining, DNA quantificiation	[44, 64–66]

*Note*. For evaluating efficiency, we used the “+” symbol, where + is the least efficient decellularization method and +++++ is the most efficient decellularization method.

**Table 6:** The evaluation of the efficiency of biological decellularization methods on corneal tissue and assays used for the evaluation.

Biological decellularization methods	Efficiency	Assays used for determination of efficiency of corneal decellularization	Sources
Enzymes	++++	Histological analysis with H&E and DAPI staining, gel electrophoresis, quantification of DNA	[7, 36, 37, 42, 47, 55, 61]
Serum	++++/+++++	Histological analysis with H&E and DAPI staining, gel electrophoresis, quantification of DNA	[61, 70, 71]

*Note*. For evaluating efficiency, we used the “+” symbol, where + is the least efficient decellularization method and +++++ is the most efficient decellularization method.
